# Biomechanical profiles of tracheal intubation: a mannequin-based study to make an objective assessment of clinical skills by expert anesthesiologists and novice residents

**DOI:** 10.1186/s12909-018-1410-0

**Published:** 2018-12-04

**Authors:** Yousuke Sakakura, Masataka Kamei, Ryota Sakamoto, Hideyuki Morii, Asami Itoh-Masui, Eiji Kawamoto, Hiroshi Imai, Masayuki Miyabe, Motomu Shimaoka

**Affiliations:** 1 Department of Clinical Anesthesiology, Mie University Graduate School of Medicine, 2-174 Edobashi, Tsu-City, Mie 514-8507 Japan; 2 Center for Information Technology and Public Relations, Mie University Hospital, 2-174 Edobashi, Tsu-City, Mie 514-8507 Japan; 3 Department of Mechanical Engineering, Mie University Graduate School of Engineering, 2-174 Edobashi, Tsu-City, Mie 514-8507 Japan; 4 Department of Emergency and Disaster Medicine, Mie University Graduate School of Medicine, 2-174 Edobashi, Tsu-City, Mie 514-8507 Japan; 5 Department of Molecular Pathobiology, Mie University Graduate School of Medicine, 2-174 Edobashi, Tsu-City, Mie 514-8507 Japan

**Keywords:** Intubation, Assessment, Technology, Motor skills, Task performance and analysis, Clinical skills

## Abstract

**Background:**

Tracheal intubation (TI) is a key medical skill used by anesthesiologists and critical care physicians in airway management in operating rooms and critical care units. An objective assessment of dexterity in TI procedures would greatly enhance the quality of medical training. This study aims to investigate whether any biomechanical parameters obtained by 3D-motion analysis of body movements during TI procedures can objectively distinguish expert anesthesiologists from novice residents.

**Methods:**

Thirteen expert anesthesiologists and thirteen residents attempted TI procedures on an airway mannequin using a Macintosh laryngoscope. Motion capturing technology was utilized to digitally record movements during TI procedures. The skill with which experts and novices measured biomechanical parameters of body motions were comparatively examined.

**Results:**

The two groups showed similar outcomes (success rates and mean time needed to complete the TI procedures) as well as similar mean absolute velocity values in all 21 body parts examined. However, the experts exhibited significantly lower mean absolute acceleration values at the head and the left hand than the residents. In addition, the mean-absolute-jerk measurement revealed that the experts commanded potentially smoother motions at the head and the left hand. The Receiver Operating Characteristic (ROC) curves analysis demonstrated that mean-absolute-acceleration and -jerk measurements provide excellent measures for discriminating between experts and novices.

**Conclusions:**

Biomechanical parameter measurements could be used as a means to objectively assess dexterity in TI procedures. Compared with novice residents, expert anesthesiologists possess a better ability to control their body movements during TI procedures, displaying smoother motions at the selected body parts.

## Background

Tracheal intubation (TI) is a core clinical skill for airway management, both in general anesthesia and in critical care medicine. Teaching the TI procedure is an important and integral part of medical education for students and residents, especially those in the anesthesiology residency training program [1]. Anesthesiologists usually learn to confidently perform the TI procedure during their residency training. How well trainees perform the TI procedure has often been subjectively evaluated by trainers (i.e., senior expert anesthesiologists). For example, direct observation by experts using semi-quantitative criteria such as checklists and/or global rating scales (GRS) has been used to evaluate trainees’ TI skills [2]. Although implementation of objective and quantitative measures to assess trainees’ clinical procedural skills would improve the quality of medical education and resident training, the development and validation of such means remain challenging.

Motion analysis represents an alternative approach for objectively assessing clinical skills and has also been used to evaluate surgical skills [3–5] in both open and laparoscopic operations. Objective parameters such as time, path length, instrument angle, number of movements, peak force, and velocity and acceleration, have been used to study the biomechanical differences between expert and novice surgical performance. Motion analysis has also been adopted for evaluating the clinical skills of anesthesiologists in performing epidural anesthesia [6], central venous catheterization [7], and transesophageal echocardiography [8]. More recently, a number of studies utilized different motion analysis technologies to evaluate TI skills [9–11]. Although these studies have identified a few differences between experts and novices, it has yet to be fully determined how dexterity in TI can be objectively assessed. An important quality of dexterity is a better ability to control body motion, thereby allowing for smoother trajectories of movement. Smoothness has been shown to reflect the hand motion dexterity of surgeons [12]. Here, utilizing a 3D-motion capture technique, we have tested the hypothesis that biomechanical parameters assessing smoothness of body motion during direct laryngoscopic TI procedures would objectively distinguish expert anesthesiologists from novice residents.

## Methods

### Subjects

The protocol of this study was reviewed and approved by the IRB of Mie University Graduate School of Medicine (Tsu-city, Mie, Japan: #3028). Two groups of subjects, 13 experts and 13 novices, were recruited (Table 1). The expert group consists of 13 board-certified anesthesiologists, while the novice group consists of 13 residents. Written informed consent was obtained from all participants.

**Table 1:** Demographic characteristics of the subjects and outcomes of the tracheal intubation attempts

	Expert (*n* = 13)	Novice (*n* = 13)	*P* value
Characteristic			
Age, mean (SD), yr.	46.9 (9.4)	27.8 (4.6)	< 0.001
Male sex, No. (%)	13 (100)	9 (69)	0.096
Height, mean (SD), cm	174 (5)	167 (9)	0.050
Experience, mean (SD), yr	22.0 (9.4)	0.39 (0.48)	< 0.001
Outcome			
First attempt success rate, %	100	100	–
Duration, mean (SD), sec			
Total duration	10.0 (2.7)	10.0 (2.2)	0.99
Phase 1 (laryngoscope)	3.9 (1.3)	3.9 (1.2)	0.94
Phase 2 (receiving tube)	1.1 (0.4)	1.1 (0.6)	0.72
Phase 3 (intubating)	5.0 (1.6)	5.0 (1.5)	0.98

### Motion capture

**Fig. 1:**
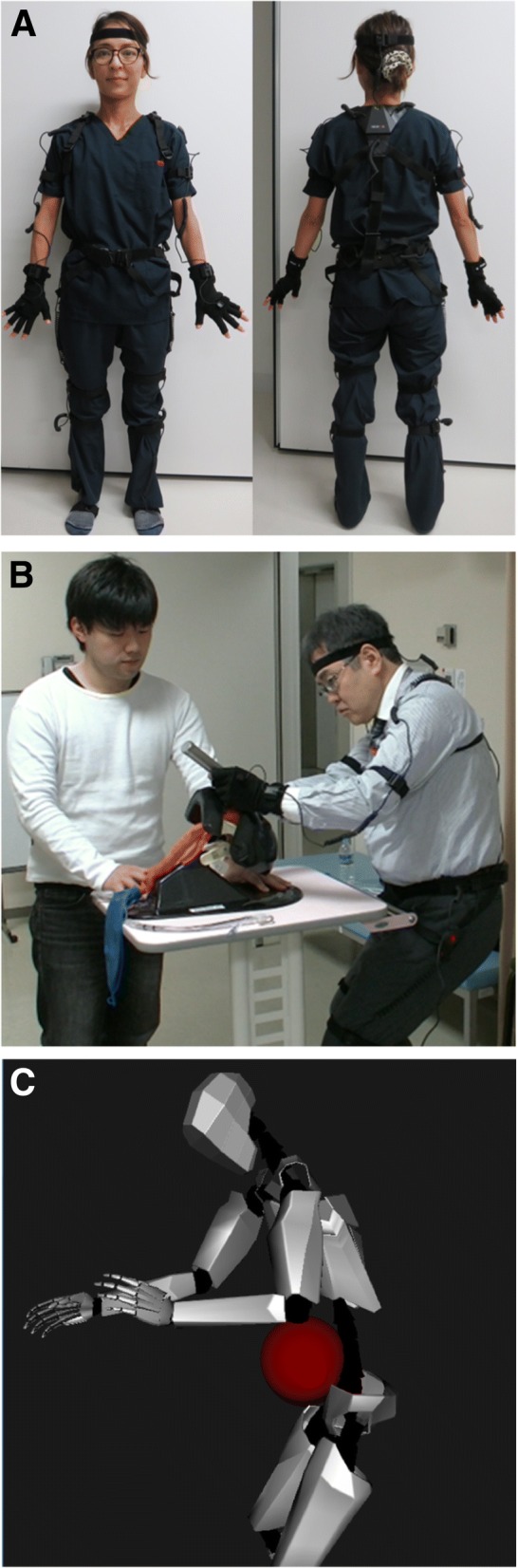
A representative photo image showing a TI attempt by a subject wearing PerceptionNeuron™ motion capturing sensors (**a**). An expert anesthesiologist performed a TI procedure on an airway mannequin while an assistant stood alongside handing him/her the TI tube (**b**). An animated image generated by the AxisNeuron™ software that represents a subject performing a TI procedure (**c**)

3D motion capture technology using a Perception Neuron™ apparatus (Noitom Ltd., Beijing, China) was employed (Fig. 1). The Perception Neuron™ apparatus comprises a series of Inertial Measurement Unit (IMU) sensor nodes that integrate a 3-axis gyroscope, a 3-axis-accerometer, and a 3-axis magnetometer to digitally record dynamic movements of the human body in 3 dimensions. AxisNeuron™ software was used in accordance with the manufacturer’s instructions to control the IMU sensor nodes in order to acquire the data and perform off-line analysis. The data streams acquired by each IMU sensor node (120 fps) placed at different parts of the subject’s body were transferred to a central hub module and stored on a computer hard disk.

### Experimental design, data collection and biomechanical parameters

Each subject wore IMU sensor nodes on his/her 21 different body parts (head, neck, right and left hands, forearms, arms, shoulders, feet, legs, upper legs, upper spine, upper middle spine, lower middle spine, lower spine, and pelvis) to encompass the movements of the entire body (Fig. 1). While wearing the IMU sensors, subjects were asked to perform a TI procedure on an adult intubation mannequin (AirSim™ Multi, TruCorp™ Ltd., Belfast, Northern Ireland, UK) using a Macintosh laryngoscope blade (size 3) and a tracheal tube (ID 8.0, TaperGurd™) installed with a stylet.

The following protocol was used to minimize variations of procedures between subjects. Each subject (expert or novice), all of whom were right-handed, stood beside the head of the mannequin and an assistant was positioned alongside him/her to hand a laryngoscope and a tracheal tube in response to the subject’s cues. Before starting, each subject placed the left hand on the face mask fitted to the mannequin and the right hand on his/her side of the body. The subject initiated his/her attempt by propping the mouth open, thus providing the assistant the first cue; specifically, to hand him/her a laryngoscope. Having received the laryngoscope with his/her left hand, each subject inserted the blade into the mannequin’s mouth, thereby carrying out a direct laryngoscopic observation of the vocal cords and glottis. When obtaining a stable laryngoscopic view of the vocal cord, each subject gave the assistant the second cue to hand him/her a tracheal tube. Holding the tube with his/her right hand, each subject then performed a tracheal intubation. Upon completion of the TI procedure, when the tube had been placed correctly, each subject gave the assistant the third cue to remove the stylet from the intubated tube, and to withdraw the laryngoscope from the mouth. After completion of the attempt, the assistant visually examined whether the TI attempt was successful or not. Each subject made 3 independent attempts to complete the TI procedure. Subjects were not allowed to practice before the attempt. All subjects had the same assistant. Body motions were recorded each time in their entirety and analyzed off-line.

**Fig. 2:**
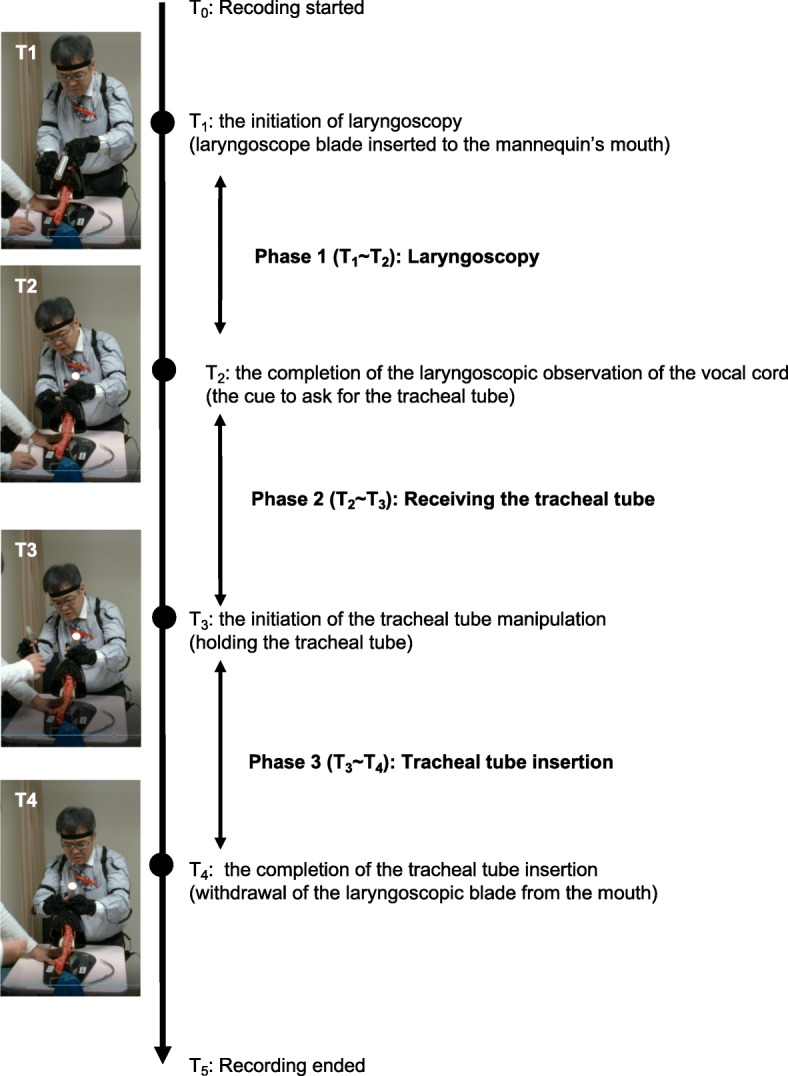
A study flow chart depicting the time sequence of a tracheal intubation attempt and illustrating the 3 phases

To analyze the dynamics of body motion during the TI procedure, time points from T_1_ to T_4_ were established and phases 1 to 3 were defined (Fig. 2). T_1_ marks the initiation of the laryngoscopy, defined as the time that the laryngoscope blade was inserted into the mannequin’s mouth. T_2_ marks the completion of the laryngoscopic observation of the vocal cords, defined as the time the second cue was given requesting the TI tube. T_3_ marks the initiation of the TI tube manipulation, defined as the total time holding the TI tube. T_4_ marks the completion of the TI insertion, defined as the time the laryngoscopic blade was withdrawn from the mouth. Phase 1 spanned T_1_ to T_2_, encompassing the primary stage of the laryngoscopy. Phase 2 extended from T_2_ to T_3_, covering the process of taking the TI tube while maintaining the laryngoscope in the correct position. Phase 3 ran from T_3_ to T_4_, encompassing the TI tube manipulation for endotracheal placement.

We focused on biomechanical parameters that assessed the subject’s ability to control his/her body motion, to include velocity (the first time derivative of position), acceleration (the second time derivative of position), and jerk (the third time derivative of position). To compare scalar values of the biomechanical parameters, mean absolute values [13, 14] for velocity, acceleration, and jerk were calculated. The mean absolute jerk measurement has been used to study skilled movements [13], as the magnitude of jerk is known to inversely correlate with motion smoothness [15].

### Statistical analysis

To perform statistical analyses, data were exported to Excel files and analyzed using SPSS software (IBM version 24). Results are presented as the mean (SD) or frequency (%) for descriptive purposes, and the mean (95% confidence interval) for inference. Normality of distribution was assessed through the use of the Shapiro-Wilk test. Logarithmic transformation of the variables was performed as needed to improve normality. Differences in characteristic data and TI attempt outcomes between groups were compared using the t test and Fisher’s exact test as appropriate. Instantaneous velocity and acceleration were directly measured at 120 fps with the Perception Neuron™ apparatus. Two-way repeated-measures analysis of variance (ANOVA) was used to compare the groups with respect to changes in mean absolute velocity, acceleration, and jerk values. Contrast tests were used if there was a significant interaction between groups and absolute value changes over time. An unpaired or Welch’s t test was used to identify which phase differences accounted for the significant *P* value when ANOVA showed a significant difference between groups. Receiver operating characteristic curves (ROCs) were constructed and the area under the curve (AUC) was assessed to compare the predictive ability for discriminating between experts and novices. Statistical significance was defined as *P* < 0.05 (two-tailed).

## Results

### Demographic characteristics of subjects and outcomes of TI attempts

The participants in the expert group, who are all board-certified members of the Japanese Society of Anesthesiologists, were significantly older and more experienced in clinical anesthesia practice than those in the novice group (Table 1). Notably, the mean height of the expert group was broadly comparable with that of the novice group (Table 1).

The TI attempts by 13 experts and 13 novices were all successful (Table 1). Overall durations of the TI attempts were indistinguishable between the expert and novice groups (Table 1). No significant inter-group differences were seen in the durations of Phase 1, Phase 2, or Phase 3 (Table 1).

### Biomechanical parameter analysis of body motions

We compared the biomechanical parameters of experts and novices during the TI attempts by studying the mean absolute values of velocity, acceleration, and jerk at IMU sensor nodes, which examined 21 different body parts during the 3 TI phases. Although the biomechanical parameters at many of the 21 body parts were indistinguishable between the expert and the novice groups, we observed consistent and statistically significant inter-group differences in selected parameters, specifically those related to the head and the left hand, as discussed below.

**Fig. 3:**
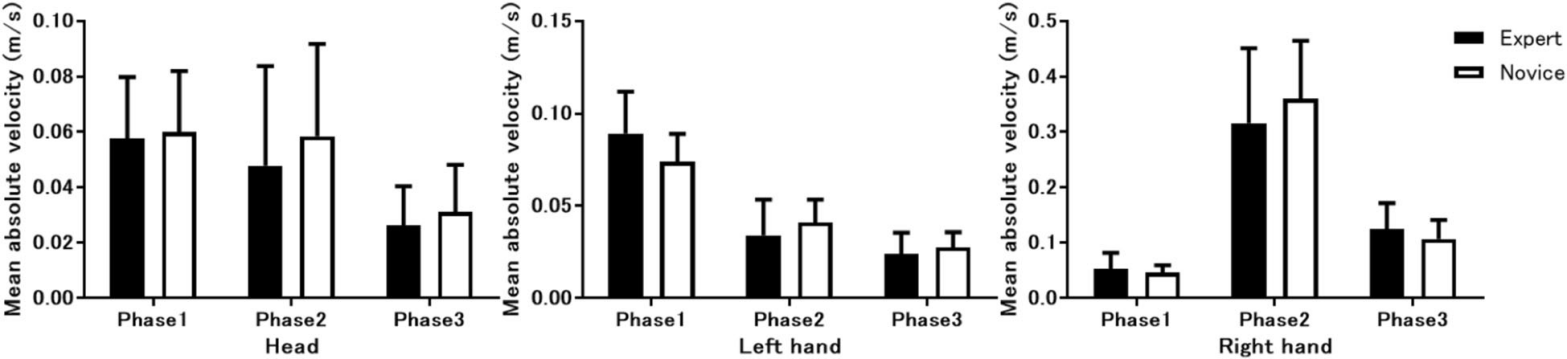
Mean absolute velocity measurements. Comparison of the mean absolute velocity measurements of body movements between expert anesthesiologists and novice residents at the head (left), left hand (middle), and right hand (right) during 3 tracheal intubation phases

**Fig. 4:**
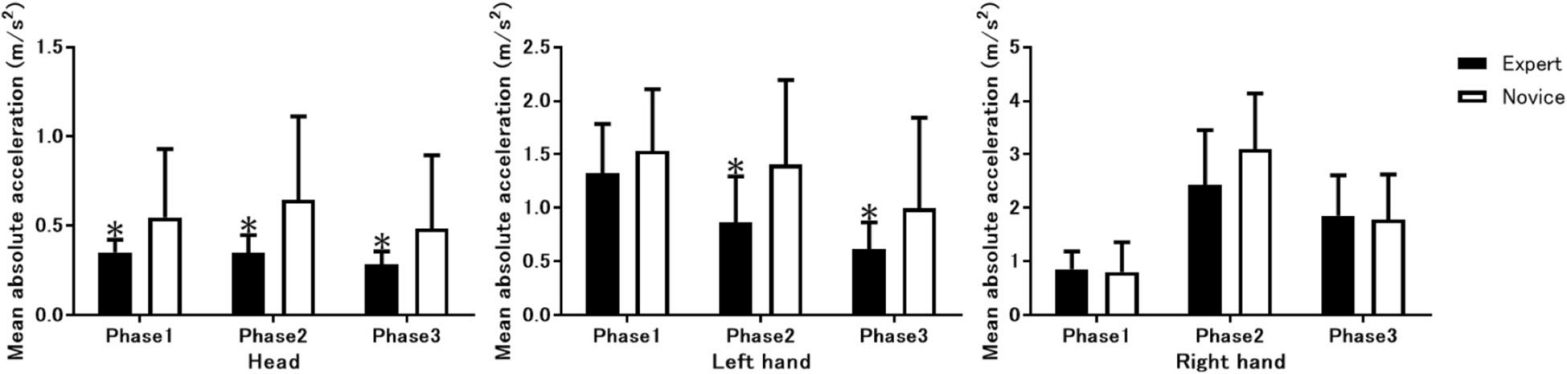
Mean absolute acceleration measurements. Comparison of the mean absolute acceleration measurements of body movements between expert anesthesiologists and novice residents at the head (left), left hand (middle), and right hand (right) during 3 tracheal intubation phases. *p < 0.05 vs. novice

Although mean absolute velocity values between experts and novices were indistinguishable at all 21 body parts (Fig. 3 and not shown), the mean absolute acceleration values at the head and the left hand, but not at the right hand nor at the other 18 body parts, were significantly lower in experts than novices (Fig. 4). At the head, mean absolute acceleration was significantly lower in the experts than the novices during all 3 TI phases. By contrast, at the left hand, mean absolute acceleration was significantly lower in the experts than the novices during Phases 2 and 3, but not Phase 1. During Phase 2, acceleration with the right hand tended to be lower in the experts than in the novices.

**Fig. 5:**
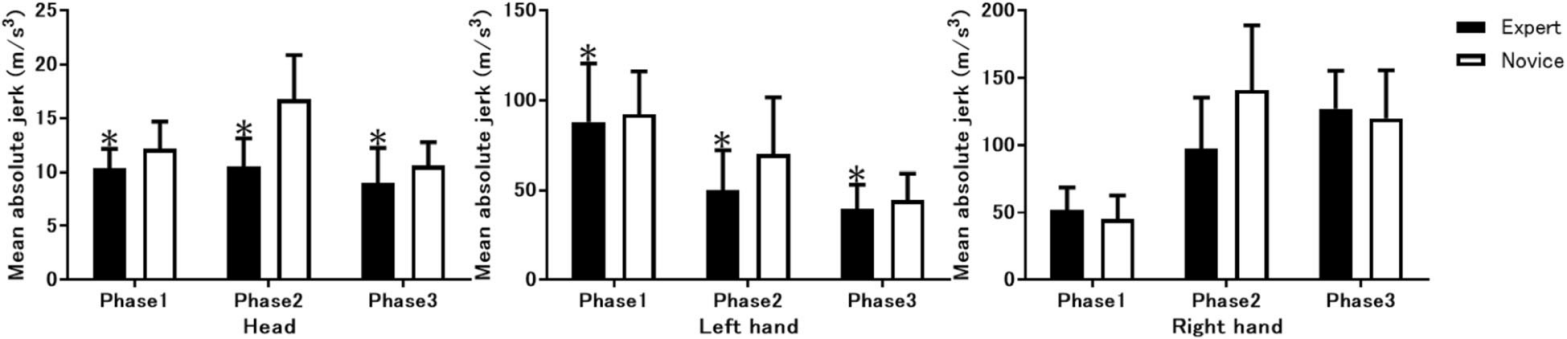
Mean absolute jerk measurements. Comparison of the mean absolute jerk measurements of body movements between expert anesthesiologists and novice residents at the head (left), left hand (middle), and right hand (right) during 3 tracheal intubation phases. *p < 0.05 vs. novice

Furthermore, the mean absolute jerk values were significantly lower in the experts than novices at the head and the left hand, but not at the right hand and other 18 body parts (Fig. 5). With the expert group both the head and the left hand showed significantly lower values, in terms of mean absolute jerk, than the novice group during all 3 phases.

### ROC analysis

Our 3D motion analysis for comparing TI procedures between experts and novices had thus far identified significant differences in the mean absolute acceleration and jerk values at the head and the left hand (Figs. 4 & 5). These results provided us with a candidate list of biomechanical parameters that could objectively distinguish skill levels in TI procedures between experts and novices. To further study the predicting ability of these biomechanical parameters, we carried out an ROC analysis of the mean absolute acceleration and mean absolute jerk values at the head and the left hand (Table 2). The biomechanical parameters that showed statistically significant predicting ability in distinguishing the skill levels of experts versus novices included the following (Table 2): the mean absolute acceleration of the head during Phases 1 to 3; the mean absolute acceleration of the left hand during Phases 2 and 3; the mean absolute jerk of the head during Phases 1 to 3; and the mean absolute jerk of the left hand during Phases 1 to 3. Among those parameters that proved positive, the mean absolute jerk of the head in Phase 2 showed an exceptionally high AUC value of 0.92, thereby suggesting a high predictive ability to distinguish between experts and novices.

**Table 2:** Area under the receiver-operating-characteristic curve for the biomechanical parameters to distinguish between experts and novices

	AUC (95% CI)	*P* value
Mean absolute acceleration		
Head		
Phase 1	0.76 (0.65–0.86)	< 0.001
Phase 2	0.83 (0.74–0.93)	< 0.001
Phase 3	0.72 (0.60–0.84)	0.001
Left hand		
Phase 2	0.76 (0.66–0.87)	< 0.001
Phase 3	0.72 (0.60–0.83)	0.001
Mean absolute jerk		
Head		
Phase 1	0.72 (0.61–0.84)	0.001
Phase 2	0.92 (0.86–0.98)	< 0.001
Phase 3	0.76 (0.65–0.86)	< 0.001
Left hand		
Phase 1	0.60 (0.47–0.73)	0.128
Phase 2	0.70 (0.59–0.82)	0.002
Phase 3	0.64 (0.52–0.77)	0.032

Only those biomechanical parameters that reached statistical significance in Figs. 1-3 are shown. Phase 1, laryngoscopy; Phase 2, receiving tube; Phase 3, intubating;

AUC, area under the receiver-operating-characteristic curve; CI, confidence interval.

## Discussion

Our study, which employed powerful 3D-motion capture technology, has shown that the expert anesthesiologists potentially exhibited significantly lower acceleration values at the head and the left hand than the residents. In addition, the expert anesthesiologists showed significantly reduced jerk values than the residents, thereby revealing that the former commanded potentially smoother motions both with the head and left hand. The ROC curves analysis demonstrated that the acceleration and jerk measurements at select body parts (i.e., head and left hand) serve as an excellent means to distinguish between experts and novices.

Previous studies employed different types of motion analysis techniques for quantitatively evaluating TI procedures, and noted certain other factors capable of distinguishing experts from novices. These included: the time and path length of the procedure [10], the laryngoscope handle angle [10, 16] and plane [9, 10], and wrist postures [11]. Specifically, Carlson et al. [9] utilized a marker-based motion capture technique, in which reflective markers were placed on the subject, the handle of the laryngoscope, and the mannequin, and the motions of the makers during the TI procedure were recorded with a near-infrared camera. A total of 3 subjects - an expert anesthesiologist, a resident, and a novice student - were included. While the path length of the left hand did not significantly differ between the expert or the intermediately experienced, and the novice, the plane of the laryngoscope was significantly different among the three. Rahman et al. [10] used a wired receiver-based electromagnetic technology to track the motions of the laryngoscope. A total of 22 subjects, 11 nurse anesthetists or anesthesiologists (expert group) and 11 medical students (novice group) were included. TI procedures with infant airway mannequins were recorded and the analysis yielded an interesting result: the experts had longer times and path lengths from insertion to withdrawal of the laryngoscope. The authors discussed the possibility that the experts might have taken more time to establish a gentle positioning. While we have not performed a head-to-head comparison of the acceleration- and jerk-measurements with other previously reported measurements such as path length [10], laryngoscope plane angles [9, 10], or wrist postures [11] on their ability to distinguish experts from novices, this question warrants further investigation.

The integration of motion analysis into multimodal feature analysis has long been sought. De Laveaga et al. [11] employed a dual-axis goniometer and torsiometer to track the wrist posture of the left arm in tandem with a surface electromyography to monitor muscle activity. A total of 20 subjects, 5 expert emergency medicine physicians and 15 medical students, were included. The study found that the experts and novices showed significantly different wrist postures and muscle utilization. Muscle activity during the TI procedures was also investigated by two other preliminary studies [17, 18] that combined motion capturing techniques with electromyography. In addition, Garcia et al. [19] measured the force applied to the blade during TI, and found that the force levels inversely correlated with the levels of expertise. The comprehensive integration of multiple factors from multimodal feature analysis [18], including motion capture techniques, electromyography, and so forth would not only facilitate a better method for distinguishing experts from novices, but would also increase the complexity of the prediction algorithm. A future advancement in computation could help manage such complex prediction algorithms. However, one might prefer a simpler formula to distinguish the performances of experts and novices, which begs the question of which single factor would best predict expert performance in TI procedures.

Higher consistency in motion trajectory in expert laryngoscopy has been proposed by Delson et al. [20, 21], who utilized a 3-dimensional force/torque sensor attached to the laryngoscope to examine patients. Our mannequin-based body motion analysis focused on velocity, acceleration, and jerk. Although trajectory path length and patterns were not our primary focus, we do acknowledge the potential importance of these parameters due to the fact that they have been extensively studied previously in both TI and surgery settings. In fact, these studies have often found large variations from subject to subject, thereby demonstrating less significant power to distinguish between expert and novice performance levels. As our study aims to establish proof-of-principle using an airway mannequin to study jerk motions in expert TI performances, further validations are needed in the near future.

We believe that less accelerated and less jerky (i.e., smoother) motions would better measure the ability of experts to control body movements during TI procedures. Reduced mean absolute jerk values have been used to show the motion smoothness associated with improved motor control tasks by students in manipulating a gaming controller [13]. Reduced jerkiness or increased smoothness has been thought to reflect coordinated movements [22]. Jerk-based measurements have also been used in studying the motion smoothness associated with better skills in manipulating medical instruments such as surgical forceps [12] and catheters for transcatheter aortic valve implantations [23]. In these studies [12, 23], the medical instruments were directly manipulated by the hand(s) of the test subjects; thus, smoother movements of the instruments should directly correlate with the coordinated movements of the hands and arms. We have demonstrated that, compared with novices, experts showed less accelerated and less jerky movements of not only the hand holding the medical instrument (the left hand holding the laryngoscope), but also the head as well. Although we did not track the subject’s gaze in the present study, less accelerated and less jerky movements of the head are likely to be associated with stable views of the larynx, thereby facilitating easy placement of the tube through the vocal cord. Visual perception and cognitive skills are integral components of expert performance in medicine and sports [24]. This study suggests that stable head movements might reflects improved perceptual skills. Similar to what has been shown in sports and in surgical training [25], an expert anesthesiologist’s performance in TI procedures would require enhanced perceptual, cognitive, and fine motor skills.

The expert and novice groups in this study showed comparable TI outcomes including the same TI success rates (i.e., 100%) and similar durations in completing TI attempts. In contrast to the previous studies [9, 10] that used reference groups consisting of students and that reported significantly different completion times in the TI attempts between expert and reference novice groups, the reference group included in the current study consisted of residents who had little, though some, experience in performing TI procedures. This study design might potentially raise the bar for distinguishing experts from non-expert references by using simple outcome-based metrics. Nevertheless, using a motion-analysis-based metrics system that examined the process, but not the outcome of the TI attempts, this study has shown that biomechanical parameter (i.e., mean absolute acceleration and jerk values) are robust enough to distinguish between expert anesthesiologists, who have substantial TI experiences, and novice residents who have limited TI experience. The robustness of the mean absolute acceleration and jerk measurement to differentiate between experts and novices is further substantiated by the fact that the variations of these values within groups were not significant (data not shown). Of note, the ROC analysis also suggested that the mean absolute jerk values of the head might be a more robust biomechanical parameter to objectively distinguish experts from novices in the TI procedures.

An intriguing question is whether the changes in the biomechanical parameters we observed represent one of the causative factors promoting expert skillfulness or merely a consequence of the skillfulness acquired by the established experts. In many domains, becoming an expert is a multifactorial process that requires not only fine motor skills, but also enhanced visual, perceptual and cognitive abilities. Thus, it would be too optimistic to state that achieving less jerky movements of the head and hand would lead to improvements in all factors towards becoming an expert. A plausible explanation would be that the less accelerated and less jerky (i.e., smoother) movements we observed are primarily a consequence of the acquired expertise. A better ability to control body motion might be linked to a high sense of confidence in expert performance.

A critical question to address in understanding the results of the present study is how educators in the field of clinical medicine could benefit from the knowledge and technology described here. One might wonder what significant roles, if any, the biomechanical parameter measurements could play in ensuring a rigorous evaluation of the clinical skills necessary to achieve a competency-based clinical education and training program. Compared with current best practice – i.e., direct observation by teachers with criteria such as a check list or global rating scales (GRS) [2] – an advantage of the biomechanical parameter measurements would be more objective and quantitative assessments for trainee feedback. By contrast, a limitation of biomechanical parameter measurements is that they evaluate the process rather than outcome of the procedure [2]. Thus, although we believe that reduced values for the jerk measurements would likely indicate a better ability to control body motion during TI procedures, they do not necessarily guarantee that the procedure was performed well. Therefore, biomechanical parameter measurements would likely be useful if combined with the checklist- or GRS-based evaluation approach, as has been suggested with the use of the prototype motion analysis technology in medicine known as ICSAD (Imperial College Surgical Assessment Device) [2]. Future investigations would need to compare the correlation of biomechanical parameter measurements with the checklist- or GRS-based approach in evaluating the competency of the TI procedures, not only mannequin simulators, but also in patients. The results of such future investigations might support the possibility that biomechanical parameter measurements could be used independently to make an objective assessment of TI skills.

Other potential limitations of this study include: 1) sample size; 2) effects of wearable sensors to restrict body movements; and 3) artificial situation of the mannequin-based study. First of all, as the sample size of this study (i.e.; 13 experts and 13 novices) was relatively small, we aware the possibilities that the demographic and physical characteristics of the subjects in this study might not necessarily reflect the diversity of trainers and trainees in medical schools and teaching hospitals. For example, all subjects in this study were right-handed who used a conventional laryngoscope designed to be handled by the left hand. Inclusion of left-handed subjects would be needed in the future investigations. Second, wearing the IMU sensor nodes might physically impede the body movements of the subjects, although the wearable equipment including all 21 sensors weigh only ~ 300 g. Wearable sensor-free motion capture approaches that employ computer-based image analysis [26] might be an alternative that would complement our results. Third, the environment that we performed our mannequin-based experiments was different from that of the operating rooms, thereby potentially eliciting any artifacts in the performances of the subjects. Future investigations involving patients administered general anesthesia in operating rooms would be necessary to clinically validate the results of the present study.

## Conclusions

The present study supports the idea that expert anesthesiologists possess a better ability to control their body motions during TI procedures than novice residents. Biomechanical parameter measurements by 3D-motion analysis might be used as a means to objectively evaluate clinical skill improvement in TI procedures, thereby strengthening the current checklist- or GRS-based evaluation approach.

## Abbreviations

AUC: Area Under the Curve
IMU: Inertial Measurement Unit
ROC: Receiver Operating Characteristic
TI: Tracheal intubation
